# Diffusiophoresis of a Weakly Charged Dielectric Fluid Droplet in a Cylindrical Pore

**DOI:** 10.3390/mi16060707

**Published:** 2025-06-13

**Authors:** Lily Chuang, Sunny Chen, Nemo Chang, Jean Chien, Venesa Liao, Eric Lee

**Affiliations:** Department of Chemical Engineering, National Taiwan University, Taipei 10617, Taiwan

**Keywords:** diffusiophoresis, droplet, cylindrical pore, boundary confinement effect, double-layer polarization, solidification phenomenon, liposome, cell, microfluidics

## Abstract

Diffusiophoresis of a weakly charged dielectric droplet in a cylindrical pore is investigated theoretically in this study. The governing fundamental electrokinetic equations are solved with a patched pseudo-spectral method based on Chebyshev polynomials, coupled with a geometric mapping scheme to take care of the irregular solution domain. The impact of the boundary confinement effect upon the droplet motion is explored in detail, which is most profound in narrow channels. We found, among other things, that the droplet moving direction may reverse with varying channel widths. Enhanced motion-inducing double-layer polarization due to the presence of a nearby channel wall is found to be responsible for it. In particular, an interesting and seemingly peculiar phenomenon referred to as the “solidification phenomenon” is observed here at some specific critical droplet sizes or electrolyte strengths in narrow channels, under which all the droplets move at identical speeds regardless of their viscosities. They move like a rigid particle without the surface spinning motions and the induced interior recirculating vortex flows. As the corresponding shear rate is zero at this point, the droplet is resilient to undesirable exterior shear stresses tending to damage the droplet in motion. This provides a helpful guideline in the fabrication of liposomes in drug delivery in terms of the optimal liposome size, as well as in the microfluidic and nanofluidic manipulations of cells, among other potential practical applications. The effects of other parameters of electrokinetic interest are also examined.

## 1. Introduction

When a solute concentration gradient is established somehow in a solution system, the solutes will migrate downward this concentration gradient, as predicted by the famous Fick’s law [1], whether the solutes are electrolytes or non-electrolytes. A corresponding osmosis flow is thus generated in the solution, referred to as the diffusioosmosis flow [2]. When this flow runs into a colloidal entity suspended in the solution, it will set the colloidal entity in motion, referred to as the diffusiophoretic motion or diffusiophoresis, a very important phoretic motion driven by the solute concentration gradient nearby. As the colloidal entities are generally charged and suspended in electrolyte solution, it is the ionic solutes in an electrolyte solution that are of major interest in practice and hence chosen as the scope of the current study.

Compared with the well-known electrophoresis, where the colloidal entity is driven by an external electric field applied upon the electrolyte solution, diffusiophoresis is a lesser-known electrokinetic phenomenon but has been gaining increasing attention in recent years due to its unique features, such as there is no or a nearly negligible Joule heating effect. In addition, other gradient-driven mechanisms, such as dielectrophoresis (motion driven by electric field gradients) [3,4,5,6], electrowetting (motion induced by surface tension gradients) [7,8,9,10], and optical trapping (motion driven by optical gradients) [11,12,13], also play significant roles in manipulating colloidal entities in various applications. However, diffusiophoresis stands out in particular in biomedical applications like drug delivery in the human body, as an increase of four degrees Celsius is fatal to mammal cells in general, and it would be impractical, if not impossible, to apply an external electric field upon the human body to manipulate the motion of the nanomedicines toward the desired region, as it is often not known in advance and the human body is too complicated, so there is no way to follow the precise route of the nanomedicine there. Diffusiophoresis, on the other hand, is capable of reaching the region needing therapy on its own. This is because the injured or sick tissue in the human body often releases specific chemicals into the body fluid and establishes a local solute concentration gradient nearby, which attracts and guides the nanomachines to its neighborhood like a cruise missile following the guiding signals to its desired area. Moreover, diffusiophoresis has been utilized in enhanced oil recovery (EOR) as well, where the remaining crude oil droplets in the porous reservoir are driven out successfully via the solute concentration gradient induced by the injection of seawater, an electrolyte solution of NaCl. Moreover, many novel applications have been reported in recent years utilizing the diffusiophoresis mechanism, such as using diffusiophoresis as an electrokinetic mechanism for particle separations in microfluidic and nanofluidic operations [14,15,16,17,18].

The nanomedicines in drug delivery are often in the form of liposomes, where the therapeutic medicines are dissolved in the fluid filling the droplet with a lipid bilayer as the droplet surface. The migration of crude oil droplets in EOR certainly is a perfect example of droplet diffusiophoresis [14]. Note that a rigid particle and a gas bubble are just limited examples of droplets with extremely high or low viscosities, respectively. Thus, the exploration of droplet phoretic motions will provide insights and valid information for rigid particles and gas bubbles as well. On the other hand, the cylindrical pore is widely used in conventional capillary electrophoresis in DNA sequencing [19,20,21,22], protein analysis [23,24,25,26], and so on, due to its convenience in operations and efficiency in temperature control to reduce the impact of the Joule heating effect, among other things. As a matter of fact, phoretic motions of droplets have been frequently encountered in microfluidic and nanofluidic operations due to their various merits [27,28,29,30,31]. The internal flow within the droplets helps mix the drugs efficiently, which can effectively reduce the cost of drug formulation. However, diffusiophoresis in general is a peculiar and lesser-known phoretic motion compared with its well-known cousin electrophoresis, which is very simple and intuitive in predicting its motion. The droplet diffusiophoresis in particular is more complicated due to its spinning surface and induced internal recirculation flow in contrast to a rigid particle. The corresponding motion in a narrow channel is even more complicated due to the boundary confinement effect upon the motion-inducing double-layer polarization. The enhanced electrostatic driving force upon the droplet is coupled simultaneously with the acceleration of the osmosis fluid flow nearby within a narrow channel. As a result, it is difficult to come up with a simple rule of thumb. As a result, we decided to conduct a theoretical study on the diffusiophoretic motion of a dielectric droplet in a cylindrical pore in particular to understand the underlying electrokinetic mechanisms leading to the ultimate droplet motion and find out the key parameters one can use to manipulate the droplet motion as desired. In other words, we extended the exploration to investigate the corresponding capillary diffusiophoresis. This classic geometric configuration certainly indicates its direct applications in micro-/nanofluidic operations. As for the droplet of particular interest in drug delivery, liposomes are often in the size range from 20 nanometers to 50 nanometers in practice or even larger sometimes [32,33]. On the other hand, capillaries with a diameter of 0.2 micron are commercially available nowadays, and pore sizes as small as 5 nanometers can be fabricated in the lab [34,35]. Hence, the boundary confinement effect, both hydrodynamic and electrokinetic, due to the presence of a nearby channel wall in a very narrow pore, has to be reckoned with carefully. As a result, this will be the focus of this study to investigate its impact upon the droplet motion, among other parameters of electrokinetic interest, such as the droplet size, the electrolyte strength, the radius of the cylindrical pore, and so on. In addition, it should be noted that a colloid in the cylindrical pore has been used as a way to model the migration of a colloid through a porous medium, with the virtual pore size evaluated from the porosity of the medium [36]. Thus, the findings in the current study are applicable to the diffusiophoresis in a porous medium as well.

Phoretic motions of a colloidal entity in a cylindrical pore have been investigated theoretically by many research groups [37,38,39,40,41,42,43,44,45,46,47]. Most of them focus on the electrophoresis phenomenon, though. The presence of a nearby cylindrical pore has been found to impose a profound boundary confinement effect upon colloidal motion in general. The hydrodynamic drag force is increased due to the no-slip boundary condition of the cylindrical pore. A large viscous shear force is thus generated, which tends to slow down the droplet motion, for one thing. Moreover, the distribution of ions within the electric double layer, the source of the electric driving force setting the colloid in motion, can be severely altered as well if the cylindrical pore is sufficiently narrow. This, in turn, changes the electrostatic environment surrounding the droplet, hence the corresponding electric driving force. The narrower the cylindrical pore is, the more significant the boundary confinement is in general, both electrostatically and hydrodynamically. In particular, Lee and his coworkers [37] explored the electrophoresis of a weakly charged dielectric droplet in a cylindrical pore recently, which is the first time the phoretic motion of a dielectric droplet has ever been studied in a cylindrical pore. Interesting phenomena like mobility reversal were reported. For droplet diffusiophoresis in a cylindrical pore, however, there has been no report in the literature, to the best of our knowledge.

Note in particular that the shear stress upon the droplet surface generated when a droplet is in motion is very detrimental to the integrity of the droplet, because the droplet surface may be destroyed if the shear stress is beyond some critical magnitude that the surface can sustain. It has been reported that, in highly constrict blood vessels, shear stress can increase by one to two orders of magnitude locally compared to normal vessels [48,49]. This may lead to the premature collapse of a liposome in drug delivery and loss of the encapsulated therapeutic medicines before it reaches the desired region needing therapy [50,51,52]. In addition, it may cause fatal breakups of the cell membranes for living cells, such as stem cells, which conduct phoretic motions in designed separation operations or in biomedical analyses in a lab-on-chip device [53,54]. As a result, it is highly desirable to operate the necessary maneuvers far away from the situation where large shear stress upon the surface of droplets may occur, as mentioned above. It turns out that the solidification phenomenon provides a promising design guideline in the fabrication stage of droplet nanomedicines like liposomes, as it will generate ideal and resilient drug carriers to achieve the maximum therapeutic performance in the human body. The details will be elaborated on in the subsequent Section 3.

In summary, we theoretically investigate the diffusiophoretic motion of a dielectric droplet in a cylindrical pore in this study. Parameters of electrokinetic interest, like the droplet size, the electrolyte strength of the suspending solution, the ratio of the droplet viscosity to that of the ambient solution, and the ratio of the droplet radius to that of the cylindrical pore, are explored to investigate their respective effect and joint impact upon the droplet motion. In order to highlight the strong boundary confinement effect in a very narrow pore, we focus on the diffusiophoretic motion of a weakly charged dielectric droplet within a chargeless cylindrical pore.

## 2. Theory

**Figure 1:**
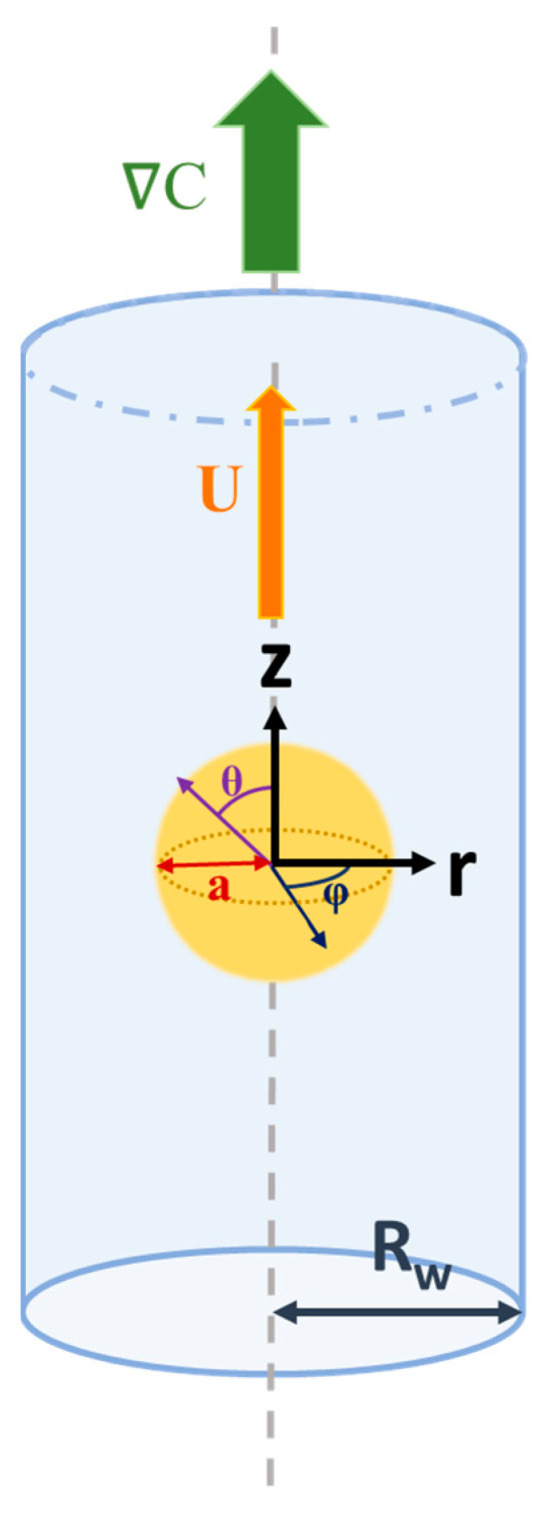
System diagram for a dielectric fluid droplet conducting diffusiophoretic motion in a cylindrical pore. ∇C is the solute concentration gradient; U is the droplet velocity; z, r, ϕ represent the cylindrical coordinates; θ, ϕ are spherical angles centered at the droplet; *a* is the droplet radius; and R_w_ is the radius of the cylindrical pore.

As shown in Figure 1, we consider the diffusiophoretic motion of a dielectric fluid droplet in response to a solute concentration gradient, ∇C, along the axis of a cylindrical pore. The cylindrical pore is filled with a binary electrolyte solution, such as KCl. The droplet is filled with chargeless dielectric fluid with a uniform surface charge distribution, assuming a constant surface charge density, which remains constant with all the varying electrokinetic parameters, such as κa, where κ is the electrokinetic strength, and a is the droplet radius. This is one of the classic surface charge conditions, in addition to the constant surface potential and the charge regulation condition [2,55]. Spherical coordinates (r, θ, and φ) are adopted with the origin located at the center of the moving droplet for the domain inside the droplet. Cylindrical coordinates (R, Θ, and Z), on the other hand, are adopted for the domain between the droplet surface and the cylindrical pore. The droplet moves along the axis of the cylindrical pore accordingly with a constant velocity U.

The electrolyte solution is assumed to be Newtonian and incompressible with constant viscosity and electric permittivity. The droplet surface is assumed to be ion-impenetrable, and it maintains its spherical shape as it moves, which is justified by the extremely low hydrodynamic Weber number, typically around 10^−7^ [56].

### 2.1. Governing Electrokinetic Equations

The governing equations are based on the general fundamental electrokinetic theory [55], which consists of the Poisson equation, the ion migration equations for both cations and anions (Nernst–Planck equation), the momentum equations for fluids both inside and outside the droplet, and the ion conservation equation for both cations and anions, as shown below:

(1) $$\nabla^{2}\phi =-\frac{\rho }{\varepsilon_{m}}$$

(2) $$\nabla^{2}\phi_{I}=-\frac{\rho }{\varepsilon_{D}}$$

(3) $$f_{j}=-D_{j}\left(\nabla n_{j}+\frac{z_{j}e}{k_{B}T}n_{j}\nabla \phi \right)+n_{j}v$$

(4) $$\nabla \cdot f_{j}=0$$

(5) $$\eta_{m}\nabla^{2}v-\nabla P-\rho \nabla \phi =0$$

(6) $${\eta_{D}\nabla }^{2}v_{I}-\nabla P_{I}=0$$

(7) ∇·v=0

(8) $$\nabla \cdot v_{I}=0$$

where Equations (1) and (2) are the Poisson equations based on Gauss’s divergence theorem for fluids both interior and exterior to the droplet, with ϕ denoting the local electrical potential and $\rho =\sum_{j=1}^{N}z_{j}en_{j}$ the space charge density, the total number of electric charges per unit volume, and N is the number of the ion species in the fluid. Moreover, n_j_ refers to the number concentration of ion species j, z_j_ is its valence number, and D_j_ is the diffusivity coefficient. Equation (3) is the ion migration equation for each ion species j, the Nernst–Planck equation. Equations (5) and (6) are the momentum equations of the fluid’s exterior and interior to the droplet, respectively, with P standing for the pressure and η_m_ and η_D_ the viscosities of the ambient solution and interior fluid, respectively. Equations (7) and (8) are the incompressibility constraints for fluids both inside and outside the droplet. In Equation (5), an additional term representing the electric body force in the surrounding electrolyte solution, −ρ∇ϕ, is included, which is the physical origin of the electric Maxwell stress tensor responsible for the local electric force. R_w_ is the radius of the cylindrical pore. In addition, ε_m_ is the electric permittivity of the ambient electrolyte solution, whereas ε_D_ is the electric permittivity of the droplet fluid. Suffix **I** overall indicates the region inside the droplet. The definitions of the rest of the symbols can be found in the List of Symbols in the Abbreviations. Note that the above governing equations are written in vector form, so are independent of the specific coordinates adopted.

Before the imposition of the solute concentration gradient, the system is at equilibrium, and a set of equilibrium solutions is obtained based on the above treatment. Once the concentration gradient is applied, the system is disturbed, and all the dependent variables will deviate from their equilibrium values. Assuming the concentration gradient is very small compared to the equilibrium concentration, these deviations will be small as well. Standard linear perturbation analysis can thus be adopted [2,57]. Assuming the variables after the disturbance satisfy the above governing equations as well, and subtracting the equilibrium equations from them, an extra set of equations governing the perturbations of the variables is obtained.

The complete governing equations are as follows:
$\nabla^{*2}\phi_{e}^{*}+\frac{{\left(\kappa a\right)}^{2}}{1+\alpha }[\exp\left(-\phi_{e}^{*}\right)-\exp\left({\alpha \phi }_{e}^{*}\right)]=0$outside the droplet(9)$\nabla^{*2}\phi_{e_{I}}^{*}=0$within the droplet(10)${\nabla^{*}}^{2}\delta \phi^{*}-\frac{(\kappa a{)}^{2}}{1+\alpha }\left[\exp(-\phi_{e}^{*})+\alpha \exp(\alpha \phi_{e}^{*})\right]\delta \phi^{*}\;=\frac{(\kappa a{)}^{2}}{1+\alpha }\left[\exp(-\phi_{e}^{*})g_{1}^{*}+\alpha \exp(\alpha \phi_{e}^{*})g_{2}^{*}\right]$
      
outside the droplet(11)$\nabla^{*2}\delta \phi_{I}^{*}=0$within the droplet(12)where δ refers to a perturbation amount of the system variable after it. Equation (9) is the governing equation for the equilibrium electric potential in the ambient electrolyte solution. Equation (10), on the other hand, indicates that there are no electric charges or ions in the interior droplet fluid. $R_{w}^{*}$ is the ratio of the cylindrical pore radius to the droplet radius, i.e., R_w_/a. Superscript * indicates dimensionless physical quantities in general, with specific definitions contained in the List of Symbols in the Abbreviations. Equations (11) and (12) are the corresponding governing equations of the electric potential disturbance based on the standard linear perturbation analysis. α is unity for the symmetric binary electrolyte solution considered here. g_j_ is the shape function showing deviation from the concentric Boltzmann distribution of ion species j when the droplet is in motion due to the effect of convection flow alone. Precise definitions can be found in the List of Symbols. Further detailed elaborations can be found elsewhere [2]. Hence,
${\nabla^{*}}^{2}g_{1}^{*}-\left(\frac{\partial \phi_{e}^{*}}{\partial r^{*}}\frac{\partial g_{1}^{*}}{\partial r^{*}}+\frac{1}{{r^{*}}^{2}}\frac{\partial \phi_{e}^{*}}{\partial \theta }\frac{\partial g_{1}^{*}}{\partial \theta }\right)\;=\frac{{Pe}_{1}}{{r^{*}}^{2}\sin\theta }(\frac{\partial \phi_{e}^{*}}{\partial \theta }\frac{\partial \psi^{*}}{\partial r^{*}}-\frac{\partial \phi_{e}^{*}}{\partial r^{*}}\frac{\partial \psi^{*}}{\partial \theta })$outside the droplet(13)${\nabla^{*}}^{2}g_{2}^{*}+\alpha \left(\frac{\partial \phi_{e}^{*}}{\partial r^{*}}\frac{\partial g_{2}^{*}}{\partial r^{*}}+\frac{1}{{r^{*}}^{2}}\frac{\partial \phi_{e}^{*}}{\partial \theta }\frac{\partial g_{2}^{*}}{\partial \theta }\right)\;=\frac{{Pe}_{2}}{{r^{*}}^{2}\sin\theta }(\frac{\partial \phi_{e}^{*}}{\partial \theta }\frac{\partial \psi^{*}}{\partial r^{*}}-\frac{\partial \phi_{e}^{*}}{\partial r^{*}}\frac{\partial \psi^{*}}{\partial \theta })$
      
outside the droplet(14)where Equations (13) and (14) are the corresponding Nernst–Planck equations governing the migration of cations and anions, respectively, in response to the solute concentration gradient. In addition,
$E^{4}\psi^{*}=-\frac{(\kappa a{)}^{2}}{1+\alpha }\left\{\begin{array}{c} \left[\frac{\partial g_{1}^{*}}{\partial r^{*}}\exp(-\phi_{e}^{*})+\frac{\partial g_{2}^{*}}{\partial r^{*}}\alpha \exp(\alpha \phi_{e}^{*})\right]\frac{\partial \phi_{e}^{*}}{\partial \theta }\;\\ -\left[\frac{\partial g_{1}^{*}}{\partial \theta }\exp(-\phi_{e}^{*})+\frac{\partial g_{2}^{*}}{\partial \theta }\alpha \exp(\alpha \phi_{e}^{*})\right]\frac{\partial \phi_{e}^{*}}{\partial r^{*}}\; \end{array}\right\}\sin\theta$outside the droplet(15)$E^{4}\psi_{I}^{*}=0$
      
within the droplet(16)where Equations (15) and (16) are the corresponding momentum equations governing the flow field outside and inside the droplet, respectively. Stream functions in dimensionless form, $\psi^{*}$ and $\psi_{I}^{*}$ are introduced to eliminate the pressure term and satisfy the incompressible constraints, Equations (7) and (8), automatically.

### 2.2. Boundary Conditions

The following are the associated boundary conditions on the droplet surface and at infinity, where the concentration gradient is imposed upon the system:
$\frac{\partial \phi_{e}^{*}}{\partial r^{*}}=-\sigma^{*}$
      
$r^{*}=1$(17)${\left.\frac{\partial \delta \phi^{*}}{\partial r^{*}}\right|}_{r^{*}=1}=0$$r^{*}=1$(18)$\frac{\partial g_{1}^{*}}{\partial r^{*}}=0$$r^{*}=1$(19)$\frac{\partial g_{2}^{*}}{\partial r^{*}}=0$$r^{*}=1$(20)${\left.\frac{\partial \Psi^{*}}{\partial r^{*}}\right|}_{r^{*}=1^{+}}={\left.\frac{\partial \Psi_{I}^{*}}{\partial r^{*}}\right|}_{r^{*}=1^{-}}$$r^{*}=1$(21)where Equation (17) indicates that the dielectric droplet under consideration here possesses constant surface charge density. Equation (18), on the other hand, indicates that the droplet is dielectric, which reduces to the electric insulating condition, i.e., the right-hand side is set to zero, if the electric permittivity of the interior fluid is much smaller than that of the ambient electrolyte solution, such as the silicone oil droplet in an aqueous electrolyte solution assumed here for simplicity. Equations (19) and (20) are derived based on the assumption that the droplet is ion-impenetrable. Equation (21) indicates that the tangent fluid velocities across the droplet surface should be continuous.
${{(\tau }^{*}}_{r\theta }^{N}+{\tau^{*}}_{r\theta }^{M}{)|}_{r^{*}=1^{+}}={(\tau^{*}}_{r\theta I}^{N}+{\tau^{*}}_{r\theta I}^{M}){|}_{r^{*}=1^{-}}$
      
$r^{*}=1$(22)

Equation (22) is the generalized Rybczynski–Hadamard condition, taking into account the electric Maxwell shear stress on the droplet surface from the exterior electrolyte solution [58]. It states that the total shear stress, including both hydrodynamic shear stress,${\tau^{*}}_{r\theta }^{N}$, and electrostatic Maxwell shear stress, ${\tau^{*}}_{r\theta }^{M}$, should be continuous across the droplet surface in the absence of interfacial tension [58,59,60]. Complete Maxwell stress tensor in general is defined as follows: $\tau^{M}=\varepsilon \left[\nabla \phi \nabla \phi -\frac{1}{2}\left(\nabla \phi \cdot \nabla \phi \right)I\right]$, where **I** is the identity tensor.

The boundary conditions at the cylindrical pore surface are listed below in dimensionless form:
$\phi_{e}^{*}=\phi_{w}^{*}=0$
      
$R^{*}=R_{w}^{*}$(23)$\frac{\partial \delta \phi^{*}}{\partial R^{*}}=0$$R^{*}=R_{w}^{*}$(24)$\frac{\partial g_{j}^{*}}{\partial R^{*}}=0$$R^{*}=R_{w}^{*}$(25)$\psi^{*}=0$$R^{*}=R_{w}^{*}$(26)$\frac{\partial \psi^{*}}{\partial R^{*}}=0$$R^{*}=R_{w}^{*}$(27)

Moreover, far away from the droplet, both upstream and downstream, the flow field and the electric field should asymptotically approach the corresponding situation in the absence of the droplet, as shown below:
$\psi^{*}=\frac{1}{2}U^{*}R^{*2}+\psi_{\infty }^{*}\left(R^{*}\right),\frac{\partial \psi^{*}}{\partial Z^{*}}=0$
      
$Z^{*}=\pm L^{*}$(28)$\phi_{e}^{*}=\phi_{e,\infty }^{*}\left(R^{*}\right)=0$$Z^{*}=\pm L^{*}$(29)$\frac{\partial \delta \phi^{*}}{\partial Z^{*}}=-\beta \nabla^{*}C^{*}=0$$Z^{*}=\pm L^{*}$(30)$g_{j}^{*}=\left(\beta -1\right)\nabla^{*}C^{*}R^{*}cos\theta =-\nabla^{*}C^{*}R^{*}cos\theta$$Z^{*}=\pm L^{*}$(31)where L*(=L/a) stands for a scaled distance large enough so that the droplet mobility does not change with the increasing L* anymore. It turns out that L* = 10 is sufficient. A dimensionless index β, defined as β $=\frac{D_{1\;}-{\;D}_{2}}{D_{1}+{\;D}_{2}},$ in a binary electrolyte solution appears in Equation (30), where D**_1_** is the diffusivity of the cations, and D_2_ is the diffusivity of the anions. β is a measurement of the strength of the induced diffusion potential [61,62]. For a KCl solution with approximately identical diffusivities of cations and anions, **β** is often regarded as zero as a benchmark situation of diffusion potential, which is set to zero here as well. Equation (31) indicates the impact of the diffusion potential upon the droplet motion, which is derived based on the electroneutrality constraint in diffusiophoresis. Here, **β** is set to zero in Equations (30) and (31) in a KCl solution. Equations (30) and (31) are the boundary conditions at infinity in general. They indicate that there should be no net electric current at infinity, since there is no external electric field applied upon the system.

Subsequent mathematical treatments and the evaluation of droplet mobility is contained in the Supplementary Materials.

## 3. Results and Discussion

**Figure 2:**
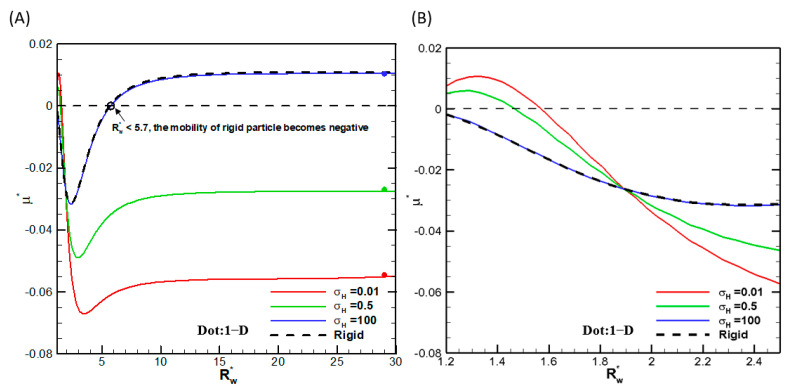
Dimensionless mobility (μ*) as a function of $R_{w}^{*}$ at various viscosity ratios σ_H_ for a dielectric droplet with σ^*^ = 2.03 and κa = 1 in the KCl solution (β = 0): (**A**) small-scale; (**B**) large-scale.

We first check the convergence of the numerical scheme in that the calculation results do not change any more with further mesh refinement. It turns out that 51 grid points in the **θ**-direction plus 100 grid points in the r-direction before conformal mapping are sufficient. We also check the convergence behavior of the droplet mobility with increasing the radius of the cylindrical pore. Indeed, they all asymptotically approach the single droplet situation in the absence of the cylindrical pore, as shown in Figure 2, where the droplet mobilities as functions of the ratio of the droplet radius to the cylindrical pore radius are shown for several viscosities of the droplet interior fluids. We thus conclude that the numerical scheme is reliable, and the calculation results are accurate. We then go on to investigate the droplet motion based on it. Note that, as shown in Figure 2, mobility reversal is observed for a highly viscous droplet, which is essentially a rigid particle with the ratio of the droplet viscosity to that of the ambient solution equal to 100. This indicates that the moving direction of a droplet in narrow channels may be opposite to a droplet in wide channels, which has potential applications for droplet manipulation in microfluidic and nanofluidic devices.

**Figure 3:**
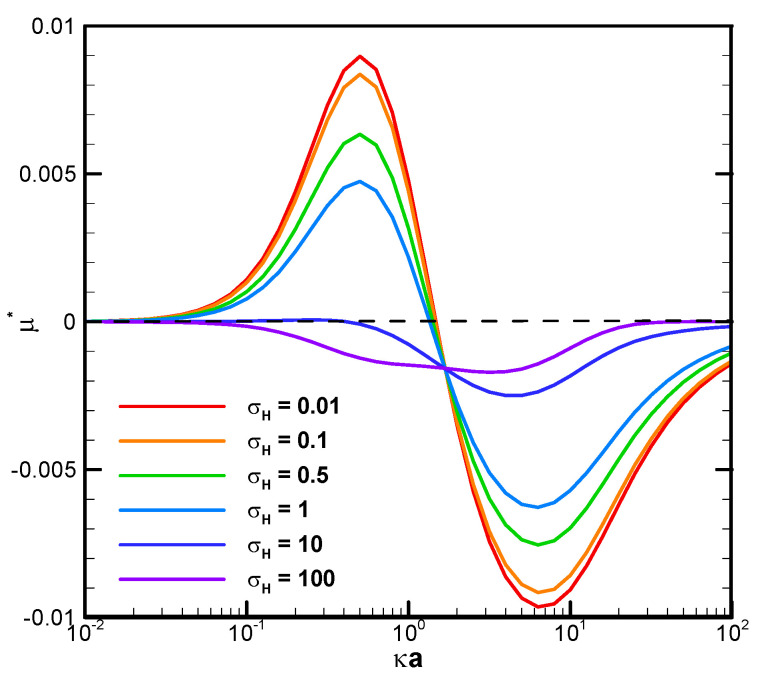
Dimensionless mobility as a function of κa with various σ_H_ for a dielectric droplet, $R_{w}^{*}$ = 1.2 with σ^*^ = 2.03 in the KCl solution (β = 0).

Figure 3 demonstrates the mobility profiles of a benchmark weakly charged droplet with the dimensionless surface charge density σ* equal to 2.03 in a very narrow cylindrical pore with $R_{w}^{*}$ = 1.2 as the function of κa for various viscosity ratios of the droplet interior fluid to that of the ambient electrolyte solution, where κ is the electrolyte strength, and a is the droplet radius. κa can be regarded as a measurement of the electric double-layer thickness. The larger the value of κa is, the thinner the electric double layer surrounding the droplet and vice versa. This is due to the double-layer compression effect when the electrolyte strength κ is high in the bulk solution. In fact, κa can be regarded as the dimensionless reciprocal of the double layer thickness, since κ^−1^, the Debye length, is a characteristic length of the double layer [55]. It is interesting to note that, for a fixed droplet size in such a narrow channel, the droplet tends to move upward against the concentration gradient in relatively dilute electrolyte solutions but downward along the concentration gradient in relatively concentrated solutions. The underlying fundamental electrokinetic mechanisms are elaborated as follows.

When a concentration gradient of ions is somehow established in an electrolyte solution, the ions will migrate downward this concentration gradient in the bulk solution by the diffusion mechanism instantly, both the cations and the anions. An osmosis flow referred to as the diffusioosmosis flow is thus generated due to the closely bonded hydrated solvent molecules moving together with the ions and the momentum transfer to the pure solvent molecules in the neighborhood via the hydrodynamic stress, as indicated by the viscosity of the solution. If the diffusivities of the cations and anions are the same, they will migrate at the same speeds. If they are different, an induced diffusion potential will be instantly generated within the bulk electrolyte solution by the Coulomb electrostatic force, which tends to speed up the slower ions and slow down the faster ones. Hence, eventually, and almost instantly, they move downward the concentration gradient in the bulk electrolyte solution at the same speeds as well. The diffusiophoresis observed in the former electrolyte solution is called chemiphoresis sometimes, emphasizing the fact that there is no induced diffusion potential involved, and the phoretic motion of the colloidal entity is purely due to the concentration/chemical affinity of the ions. In the latter case, in addition to the motion caused by the chemiphoresis mechanism, the induced diffusion potential inside the bulk electrolyte solution tends to behave as an inner battery to drive the charged colloidal entity just like an externally applied electric field. The motion induced by this mechanism is called the electrophoresis component of diffusiophoresis, emphasizing the electrokinetic origin of its driving mechanism. Note that the chemophoresis mechanism is always present with or without the electrophoresis mechanism. It is coupled with the electrophoresis component if there is indeed a diffusion potential generated in the electrolyte solution. Hence, it is sometimes referred to as the chemiphoresis component. Note that these two components are coupled and cannot be rigorously separated from each other. When these ions in the diffusioosmosis flow run into a charged droplet ahead, both cations and the anions migrate across the boundary of the electric double layer surrounding the charged droplet at the same speed and total amount. The counterions will be predominantly attracted in the upstream hemisphere of the charged droplet due to the Coulomb electrostatic attraction force between them and the droplet surface charges. The co-ions, on the other hand, will be predominantly repulsed and swept toward the downstream hemisphere instead, based on the Coulomb electrostatic repulsion force. An induced asymmetric double-layer polarization generates a local electric field driving the droplet in motion. The force balance between this electric driving force and the hydrodynamic drag force determines the ultimate droplet motion pattern. Note that, while the fundamental scenario presented above indicates initially that this electric driving force tends to drive the droplet upward against the concentration gradient based on the Coulomb electrostatic force, the actual moving direction of the droplet can go either way. Rigorous solution of the governing fundamental electrokinetic equations is the only way to sort it out, as presented in Figure 3 above.

When the ions in the downward-moving diffusioosmosis flow are relatively dilute, its associated downward pushing force is relatively weak as well. The upward electric driving force is hence dominant and pushes the droplet upward. A positive droplet mobility is thus observed, as shown in Figure 3. When κa increases further, however, the downward osmosis convection flow becomes significantly enhanced. The droplet is pushed downward eventually, and a negative mobility is observed, as shown in Figure 3 as well. This indicates an interesting phenomenon that one can manipulate the moving direction of a droplet in a narrow channel simply by changing the concentration of the electrolyte solution. Moreover, under the same geometric configuration filled with identical electrolyte solution, smaller droplets tend to move to the region with a higher concentration of ions and larger ones in the opposite direction. In drug delivery with liposome droplets, the injured tissues or regions needing therapy often release specific chemicals in their neighborhood. A concentration gradient of these chemicals is thus established, which serves as the natural guiding and driving mechanisms to lead the liposomes to their desired final destination [63,64]. Smaller liposomes would be favorable, according to Figure 3.

**Figure 4:**
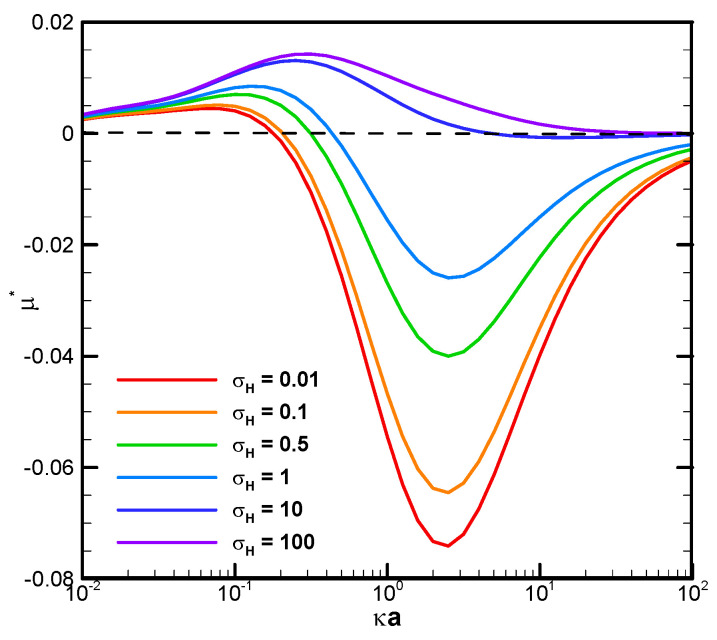
Dimensionless mobility as a function of κa with various σH for a single dielectric droplet, with σ* = 2.03 in the KCl solution (β = 0) (Reprinted from Figure 5a in Ref. [64]. Used with permission. © Physics of Fluids; 2021 AIP Publishing).

The boundary confinement effect is clearly demonstrated by direct comparison with the corresponding mobility profiles of a droplet suspended in an infinite medium of electrolyte solution, as shown in Figure 4. The droplet mobility is significantly increased at smaller κa, or a thicker double layer, due to the presence of a nearby cylindrical pore. The ion concentration in the double layer is significantly increased by this geometric confinement effect, leading to a much stronger motion-inducing double-layer polarization. The electric driving force is thus significantly enhanced, leading to a mobility behavior completely opposite to the single droplet situation. When the double layer is thin, indicated by a relatively large κa value, however, the nearby channel wall will not be able to confine the double layer as profoundly as before. The boundary confinement effect will weaken significantly. The droplet mobility profiles, hence, assume a pattern similar to the corresponding single droplet situation.

**Figure 5:**
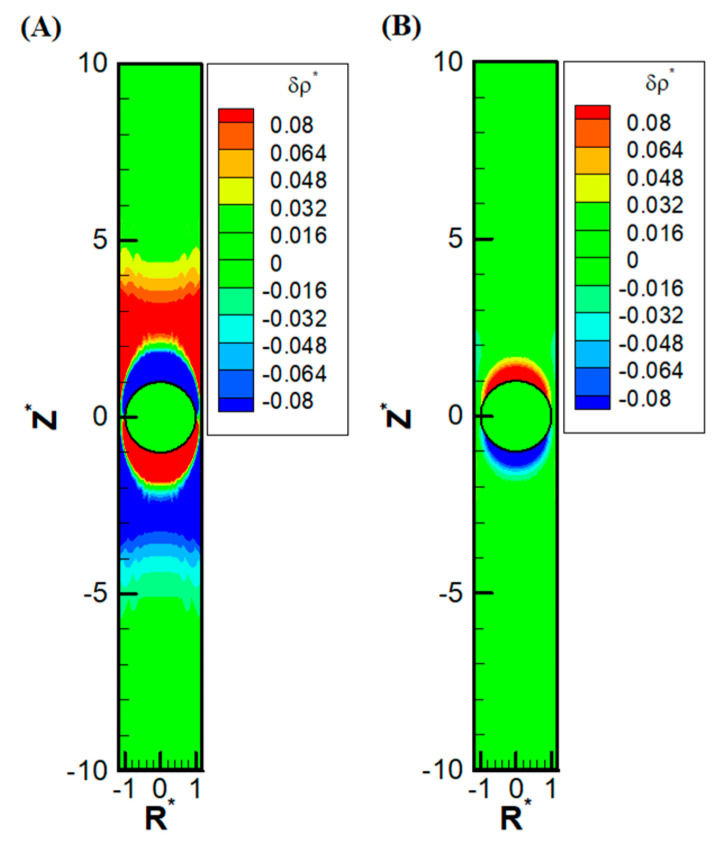
Contour plots of perturbation distribution of the dimensionless charge density (δρ^*^) with σ^*^ = 2.03, σ_H_ = 0.5, κa = 1, $and\;R_{w}^{*}$ = 1.2 in the KCl solution (β = 0): (**A**) diffusiophoresis; (**B**) electrophoresis. (R^*^ represents the radial coordinate, Z^*^ represents the axial coordinate, and $R_{w}^{*}$ represents the dimensionless cylinder radius).

Other than the obvious confinement effect upon the droplet mobility, the presence of the nearby channel wall also has a subtle impact on the ion distribution upstream and downstream of the droplet. Due to the significant reduction of area available for the downward diffusioosmosis flow at the equator area, the ions have a huge buildup in the upstream. This, in turn, induces an extra outer region of a secondary double layer based on the electrostatic Coulomb law. An extra enhancement of co-ions results downstream as well, which also induces an extra outer region enriched in counterions. Moreover, as the macro-/convection osmosis flow has to pass through the narrow annulus region at the equator, a severe buildup of ions also takes place near the channel wall, both upstream and downstream of the droplet. The above deduction based on electrokinetics is clearly observed in Figure 5A, where the precise shape of the double-layer polarization is demonstrated. Compared with the corresponding double-layer polarization in droplet electrophoresis, as shown in Figure 5B, the ion distribution is much more complicated in diffusiophoresis.

**Figure 6:**
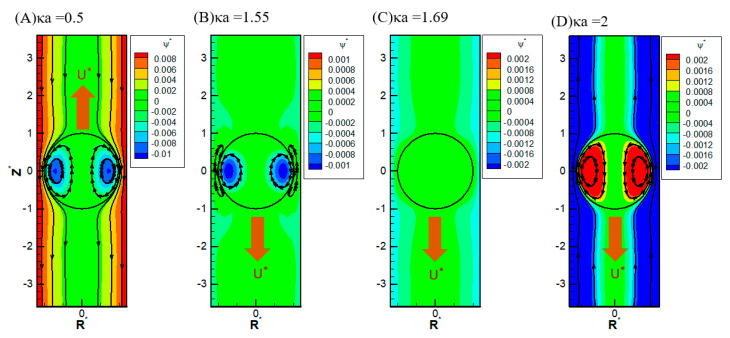
Contour plot of the stream function with σ^*^ = 2.03, σ_H_ = 0.5, $and\;R_{w}^{*}$ = 1.2 in the KCl solution (β = 0): (**A**) κa = 0.5; (**B**) κa = 1.55; (**C**) κa = 1.69; (**D**) κa = 2.

It is also interesting to note that, in Figure 3 and Figure 6C, there is a critical value of κa where all droplets move at identical speeds regardless of their viscosities. They move like rigid particles in that the characteristic recirculating interior vortex flows pertinent to the typical droplet motion completely disappear. We thus call it the “solidification phenomenon” [64,65]. This intriguing phenomenon is due to the deadlock of the spinning electric Maxwell traction and the hydrodynamic drag force on the droplet surface, which are in opposite directions here [64,65]. Further detailed electrokinetic mechanisms will be elaborated as follows by examining more droplet motion patterns, both droplets moving directions and surface spinning orientations.

It is interesting to note that, while the droplet moves upward, indicating the overall dominance of the upward electric driving force relative to the weak downward osmosis flow, the portion attributed to the spinning force on the droplet surface may still be no match for the hydrodynamic drag force spinning droplet surface downward, as shown in Figure 6A. The major portion of the electric driving force goes to the pressure drag (form drag) pushing the droplet upward as a whole by exerting the force in the normal direction of the droplet surface [58]. As a result, the remaining viscous drag (skin drag), which spins the droplet surface in a tangent direction, is insufficient in this case to reverse the original spinning orientation on the droplet surface by hydrodynamic drag force from the osmosis flow [58]. However, as shown in Figure 6B, when κa further increases to 1.4, the droplet moves downward with an axisymmetric exterior vortex flow to reconcile the two electric and hydrodynamic shear forces spinning the droplet surface in opposite directions [65]. It indicates that the orientation of the surface spinning direction cannot be decided by the moving direction of the droplet alone. They are not correlated in a simple way in general. It can only be determined by the precise relative portions of the electric driving force exerted upon the droplet surface in the form of either the pressure drag or the viscous drag upon the droplet. This distribution of relative portions is case-dependent and can only be determined by solving the governing electrokinetic equations coupled with the associated boundary conditions rigorously, as presented here. There is no simple rule of thumb for the prediction of the surface spinning direction and moving direction of a droplet, as well as its speed, for a complicated system, like the one considered here. As κa increases further, the opposite spinning forces from the electric driving force and hydrodynamic drag force become identical in magnitude eventually at a critical value of κa, and the droplet surface spins no more. Without the net surface spinning motion, the droplet now moves like a rigid particle without the interior recirculating vortex flow, which is referred to as the “solicitation phenomenon” here. As a result, the droplet mobility is independent of the droplet viscosity, as shown in Figure 6C. In other words, regardless of the droplet’s viscosity, its mobility remains the same. A further increase of κa may even lead to the complete suppression of counterclockwise electric spinning motion via the overwhelming clockwise hydrodynamic spinning force, as shown in Figure 6D.

The solidification phenomenon has important potential in practical applications like cell manipulation and drug delivery, to name a few. For animal cells and certain algal cells that lack a cell wall, for example, the cell contents are separated from the external environment only by a very thin lipid cell membrane. Cellular damage could be significantly enhanced by both the increase in intensity and the length of time over which the shear stress is applied [54]. Since the shear rate at the droplet surface is zero at the solidification point, the droplet can resist, or at least minimize, the undesirable external shear stress, thus avoiding potential cell damage during its motion. Another example can be found in typical bioreactor operations involving cells, where the vulnerability of cells to the shear stress generated by agitated mixing, among other sources, is determined by their resilience to it [53,54]. Additionally, in drug delivery with liposomes, the drug might leak during transportation because of the shear stress experienced before they reach the intended region needing therapy. This unwanted early release of the encapsulated nanomedicines can result in reduced overall drug performance and negative side effects [50,52,66]. With the solidification phenomenon pertinent to droplets, however, it provides the key information to solve all the problems mentioned above caused by the undesirable shear stress damaging to the droplet surface: the critical κa value at which the solidification phenomenon happens. For a cell of specific size, it provides a promising electrolyte strength at which the shear stress is absent if diffusiophoresis is the driving mechanism for its motion. On the other hand, for drug delivery in the human body, where the electrolyte strength is not an adjustable parameter, the optimal size of the drug-carrying liposomes in the fabrication stage can be obtained via the corresponding critical κa value, as diffusiophoresis is a crucial mechanism to enhance the concentration of drugs in the intended region of the human body [67]. The reduction in cell mortality and enhancement of the success in drug delivery is anticipated in this way. Note that the solidification phenomenon is observed in the diffusiophoresis of a highly charged dielectric fluid droplet in an infinite medium of electrolyte solutions as well [56,64]. Here, we find that it can happen for a weakly charged droplet in a narrow cylindrical pore as well. The enhancement of the motion-inducing double-layer polarization by the boundary confinement effect in narrow channels is the underlying electrokinetic mechanism.

## 4. Conclusions

In this study, the diffusiophoretic motion of a weakly charged dielectric droplet in a cylindrical pore filled with KCl electrolyte solution is investigated theoretically. The droplet contains no electrolytes inside and has a constant, uniform surface charge density. Corresponding governing fundamental electrokinetic equations are solved with a patched pseudo-spectral method based on Chebyshev polynomials, coupled with a geometric mapping scheme to take care of the irregular shape of the solution domain. The results are summarized as follows:

(1) Mobility reversal is observed in a benchmark narrow cylindrical pore. Moreover, an interesting and peculiar phenomenon referred to as the “solidification phenomenon” is observed at some specific critical droplet sizes or electrolyte strengths in narrow channels under which the droplet mobilities are identical regardless of the droplet viscosities. The droplets move like rigid particles without the surface spinning motions or interior recirculation flows. As the corresponding shear rate is zero at this point, and so is the total shear stress upon the droplet surface, the droplet is resilient to exterior damaging shear stress that tends to destroy the integrity of the droplet in motion. This provides an ideal and optimal droplet size in practical applications such as drug delivery, where the damage to the therapeutics-carrying liposomes can be minimized as a result before the nanomedicines reach the intended region of the human body. The overall performance can be maximized in this sense. For microfluidic and nanofluidic operations involving droplets, this provides a guideline for the ideal electrolyte strength of the solution filling the cylindrical pore as well if the damage to the moving droplet by surface shear stress is of major concern, such as in cell operations. This solidification phenomenon is found in a highly charged dielectric droplet conducting either electrophoretic or diffusiophoretic motion in an infinite medium of electrolyte solution as well. We show here that, with the presence of a very close channel wall, the same phenomenon can take place for a weakly charged droplet as well.
(2) While the droplet may move either up against the concentration gradient or downward with it, there is no simple correlation between its moving direction and the orientation of its surface spinning direction. It can go either way. The relative distribution between the pressure drag and the viscous drag of the electric driving force is crucial. The force balance between the electric driving force, which is induced by the double-layer polarization, and the hydrodynamic drag force from either side of the droplet surface determines the ultimate droplet motion pattern.
(3) The precise shape of the motion-inducing double-layer polarization is presented, which is much more complicated than the corresponding electrophoresis system. The convection downward diffusioosmosis flow is found to be responsible for it. Its impact is significantly enhanced in narrow channels in particular due to the speedup of the fluid flow through the narrow annular cross-section area surrounding the droplet equator. Buildup of ions upstream due to this reduction in the fluid flow area takes place as a result. Induced secondary double-layer polarization is observed under certain electrokinetic circumstances.
(4) Size-dependent mobility in a cylindrical pore is observed, which provides a potential separation scheme in nano-/microfluidic operations involving dielectric droplets of varying sizes.

Overall, the findings in this study provide a comprehensive understanding of the droplet diffusiophoresis behavior in a cylindrical pore, which is a classic geometric scheme in various practical applications involving droplets, such as drug delivery and droplet microfluidic/nanofluidic operations.

## Data Availability

Data sharing is not applicable to this article, as no datasets were generated or analyzed in the current study.

## Supplementary Materials

The following supporting information can be downloaded at https://www.mdpi.com/article/10.3390/mi16060707/s1, Figure S1: Two-dimensional physical domain; Figure S2: Mesh diagram of sub-regions; Figure S3: Orthogonal mesh diagram of sub-regions after conformal mapping; Table S1: Table of Relative Permittivity (εr) of Various Materials. References [2,57,68,69,70,71,72,73,74,75] are cited in the supplementary materials.

## Abbreviations

**List of Symbols**	
a	radius of the droplet (m)
η	viscosity (kg/m s)
C	concentration of total electrolytes (1/m^3^)
C_0_	characteristic concentration of electrolytes ions (1/m^3^)
C*	$\mathrm{dimensionless}\;\mathrm{concentration}\;\mathrm{of}\;\mathrm{total}\;\mathrm{electrolytes}\;\mathrm{defined}\;\mathrm{as}\;\frac{C}{C_{0}}$
D_j_	diffusivity of ionic species j (m^2^/s)
β	$\mathrm{dimensionless}\;\mathrm{diffusivity}\;\mathrm{difference}\;\mathrm{between}\;\mathrm{the}\;\mathrm{cations}\;\mathrm{and}\;\mathrm{the}\;\mathrm{anions}\;\mathrm{in}\;a\;\mathrm{symmetric}\;\mathrm{binary}\;\mathrm{electrolyte}\;\mathrm{solution}\;\mathrm{defined}\;\mathrm{as}\;\frac{D_{1\;}-{\;D}_{2}}{D_{1}+{\;D}_{2}}$
e	elementary charge of an electron (1.6 × 10^−19^ coul)
F_DZ_	magnitude of the hydrodynamic drag force exerted on the droplet (Nt)
F_EZ_	magnitude of the electric force exerted on the droplet (Nt)
$F_{DZ}^{*}$	dimensionless magnitude of the hydrodynamic drag force exerted on the droplet
$F_{EZ}^{*}$	dimensionless magnitude of the electric force exerted on the droplet
f_j_	molar flux of ionic species j (mole/m^2^/s)
g_j_	shape function representing the extra non-concentric concentration perturbation of ionic species j (volt)
$g_{j}^{*}$	$\mathrm{dimensionless}\;\mathrm{shape}\;\mathrm{function}\;\mathrm{representing}\;\mathrm{the}\;\mathrm{extra}\;\mathrm{non}-\mathrm{concentric}\;\mathrm{concentration}\;\mathrm{perturbation}\;\mathrm{of}\;\mathrm{ionic}\;\mathrm{species}\;j\;\mathrm{defined}\;\mathrm{as}\;\frac{g_{j}}{\phi_{0}}$
k_B_	Boltzmann constant (1.38 × 10^−23^ joul/K)
n_j_	number density of ionic species j (1/m^3^)
$n_{j}^{*}$	$\mathrm{dimensionless}\;\mathrm{number}\;\mathrm{density}\;\mathrm{of}\;\mathrm{ionic}\;\mathrm{species}\;j\;\mathrm{defined}\;\mathrm{as}\;\frac{n_{j}}{n_{10}}$
P	hydrodynamic pressure
Pe_j_	Peclet number of ionic species j (Pe_j_ = U_0_a/D_j_) representing the reciprocal of its diffusivity in a dimensionless form
T	Absolute Temperature (K)
r	r-coordinate in spherical coordinates (r, θ, φ)
$R^{*}$	dimensionless r-coordinate in spherical coordinates, defined as r/a
$R_{w}$	the radius of cylindrical pore (m)
$R_{w}^{*}$	$\mathrm{the}\;\mathrm{ratio}\;\mathrm{of}\;\mathrm{the}\;\mathrm{cylindrical}\;\mathrm{pore}\;\mathrm{radius}\;\mathrm{to}\;\mathrm{the}\;\mathrm{droplet}\;\mathrm{radius},\;\mathrm{defined}\;\mathrm{as}\;R_{w}/a$
U	diffusiophoretic velocity of the droplet under consideration
**v**	velocity vector
v_0_	$\mathrm{characteristic}\;\mathrm{velocity}\;\mathrm{in}\;\mathrm{diffusiophoretic}\;\mathrm{motion}\;\mathrm{defined}\;\mathrm{as}\;\frac{\varepsilon_{m}}{\eta_{m}a}{\left(\frac{k_{B}T}{z_{1}e}\right)}^{2}$
**v***	$\mathrm{dimensionless}\;\mathrm{diffusiophoretic}\;\mathrm{velocity}\;\mathrm{defined}\;\mathrm{as}\;\frac{v}{v_{0}}$
z_j_	charge valence of ion species j
ϕ_w_*	the electrical potential of the wall
ϕ_0_	thermal potential in a binary electrolyte solution (ϕ_0_ = $\frac{k_{B}T}{z_{1}e}$)
ρ	$\mathrm{space}\;\mathrm{charge}\;\mathrm{density}\;\mathrm{defined}\;\mathrm{as}\;\sum_{j=1}^{N}z_{j}en_{j}$ (coul/m^3^)
δρ	perturbation of the space charge density
δρ^*^	dimensionless perturbation of the space charge density
δϕ	perturbation of the electric potential
δϕ^*^	$\mathrm{dimensionless}\;\mathrm{perturbation}\;\mathrm{of}\;\mathrm{the}\;\mathrm{electric}\;\mathrm{potential}\;\mathrm{defined}\;\mathrm{as}\;\frac{\delta \phi }{\phi_{0}}$
ε	electric permittivity (coul/volt/m)
θ	θ-coordinate in spherical coordinates (r, θ, φ)
κ	$\mathrm{Debye}\;\mathrm{length}\;\mathrm{defined}\;\mathrm{as}\;\left(\sqrt{\frac{\sum_{j=1}^{2}n_{j0}{\left(ez_{j}\right)}^{2}}{\varepsilon k_{B}T}}\right)$
μ	diffusiophoretic mobility of the particle defined as U∇C
μ*	dimensionless diffusiophoretic mobility of the particle defined as μ*=μU0(ϕ0a)
σ	surface charge density (coul/m^2^)
σ*	$\mathrm{dimensionless}\;\mathrm{surface}\;\mathrm{charge}\;\mathrm{density}\;\mathrm{defined}\;\mathrm{as}\;\frac{\sigma a}{\varepsilon_{m}\phi_{0}}$
φ	φ-coordinate in spherical coordinates (r, θ, φ)
ψ	stream function
$\psi_{\infty }^{*}$	the dimensionless stream function standing for the flow field within the cylindrical pore in the absence of the charged droplet
φ	electric potential (volt)
ϕ_e∞_*	the electrical potential distribution within the cylindrical pore in the absence of the charged droplet
$\tau_{r\theta }^{N}$	the hydrodynamic stress tensor
$\tau_{r\theta }^{M}$	the electric Maxwell stress tensor
**Operators**	
**E** ^2^	$\mathrm{operator}\;\mathrm{defined}\;\mathrm{as}\;E^{2}=\frac{\partial^{2}}{\partial r^{2}}+\frac{\sin\theta }{r^{2}}\frac{\partial }{\partial \theta }\left(\frac{1}{\sin\theta }\frac{\partial }{\partial \theta }\right)$ in spherical coordinates (r, θ, φ)
**E** ^4^	**E**^4^ = **E**^2·^**E**^2^
∇	gradient operator
$\nabla^{2}$	Laplacian operator
∇	divergence operator
${\nabla_{r}^{*}}^{2}$	r-directional Laplacian operator
**Superscripts**	
*	dimensionless variable
**Subscripts**	
1	cation
2	anion
e	equilibrium state
j	j-th ionic species
