# Supplementary material for: Diffusiophoresis of a Weakly Charged Dielectric Fluid Droplet in a Cylindrical Pore
Source: Micromachines (Basel). 2025 Jun 13;16(6):707. doi: 10.3390/mi16060707 (PMC12195106; doi:10.3390/mi16060707)
Supplement: Supplementary file 1 [file micromachines-16-00707-s001.zip › micromachines-3631835-supplementary.pdf]

Supplementary Materials

A. Subsequent Mathematical Treatments and Evaluation of Droplet Mobility

Mathematical treatments

As the system is axisymmetric, there is no dependence on φ, and the problem reduces to a two-dimensional one. Moreover, as the droplet interior fluid is chargeless, there is no need to solve for the electric field there. Since there is an analytical formula for the purely hydrodynamic fluid field inside a droplet, which can be incorporated into the corresponding exterior fluid field as a boundary condition, only the electric and fluid flow fields in the exterior region need to be solved. To accomplish this, a conformal mapping technique is adopted to convert the irregular domain exterior to the droplet into three consecutive rectangular subdomains with continuity conditions of all the system variables and their derivatives assumed across the boundaries between each of these subdomains. A patched pseudo-spectral method based on Chebyshev polynomials is then applied in both of the orthogonal directions of each subdomain [1–3]. Detailed mathematical treatments can be found elsewhere [4,5]. A numerical algorithm is then developed based on the above treatments, which is used to solve for the coupled electric and flow fields of interest here.

Evaluation of Droplet Mobility

Once the coupled electric field and the flow field are solved via the numerical algorithm developed, the corresponding dimensionless hydrodynamic drag force ($F^*_{\mathbf{Dz}}$) and the electric driving force $F^*_{\mathbf{Ez}}$ upon the droplet surface can be evaluated, respectively, as follows:

$$F^*_{Dz} = \pi \int_0^{\pi} \left| r^{*4} \sin^3\theta \frac{\partial}{\partial r^*}\left(\frac{E^{*2}\psi^*}{r^{*2}\sin^2\theta}\right)\right|_{r^*=1} d\theta - \pi \int_0^{\pi} \left| r^{*2}\sin^2\theta \left\{ \frac{(\kappa a)^2}{1+\alpha} [\exp(-\phi^*_e)(1 - \delta\phi^* - g^*_1) - \exp(\alpha\,\phi^*_e)(1 + \alpha(\delta\phi^* + g^*_2))] \frac{\partial \phi^*_e}{\partial\theta} + [\exp(-\phi^*_e) - \exp(\alpha\,\phi^*_e)] \frac{\partial \delta\phi^*}{\partial\theta} \right\} \right|_{r^*=1} d\theta \quad \text{(S1)}$$

$$F^*_{Ez} = \pi \int_0^{\pi} \left| \left\{ r^{*2}\sin\theta\cos\theta \left[\left(\frac{\partial \phi^*_e}{\partial r^*}\right)^2 + 2\frac{\partial \phi^*_e}{\partial r^*}\frac{\partial \delta\phi^*}{\partial r^*} \right] + r^{*2}\sin^2\theta \left(\frac{\partial^2 \phi^*_e}{\partial r^*\partial\theta}\frac{\partial \phi^*_e}{\partial r^*} + \frac{\partial^2 \phi^*_e}{\partial r^*\partial\theta}\frac{\partial \delta\phi^*}{\partial r^*} + \frac{\partial \phi^*_e}{\partial r^*}\frac{\partial^2 \delta\phi^*}{\partial r^*\partial\theta} + \frac{1}{r^{*2}}\frac{\partial \phi^*_e}{\partial\theta}\frac{\partial^2 \phi^*_e}{\partial\theta^2} + \frac{1}{r^{*2}}\frac{\partial^2 \phi^*_e}{\partial\theta^2}\frac{\partial \delta\phi^*}{\partial\theta} + \frac{1}{r^{*2}}\frac{\partial \phi^*_e}{\partial\theta}\frac{\partial^2 \delta\phi^*}{\partial\theta^2} \right) - 2r^*\sin^2\theta \left(\frac{\partial \phi^*_e}{\partial r^*}\frac{\partial \phi^*_e}{\partial\theta} + \frac{\partial \phi^*_e}{\partial r^*}\frac{\partial \delta\phi^*}{\partial\theta} + \frac{\partial \delta\phi^*}{\partial r^*}\frac{\partial \phi^*_e}{\partial\theta} \right) \right\} \right|_{r^*=1} d\theta \quad \text{(S2)}$$

The droplet diffusiophoretic mobility, defined as droplet velocity divided by the magnitude of the concentration gradient imposed, can be evaluated as follows:

$$\boldsymbol{\mu}^* = \frac{\mathbf{U}^*}{\boldsymbol{\nabla}^* C^*} = -\frac{\mathbf{F}^*_{\mathbf{E}}\ (\text{obtained in sub-problem two})}{\mathbf{F}^*_{\mathbf{D}}\ (\text{obtained in sub-problem one})} \quad \text{(S3)}$$

where the ingenious methodology by O’Brien and White [6] is adopted, which demonstrates that the system can be decomposed into two separate artificial auxiliary sub-problems, with one of them corresponding to a purely electrostatic one, sub-problem one, and the other a purely hydrodynamic one, sub-problem two.

B. Domain Decomposition and Patched Pseudo-Spectral Method Adopted here

The axisymmetric nature of the problem reduces it to a two-dimensional half-domain, as shown in Figure S1:

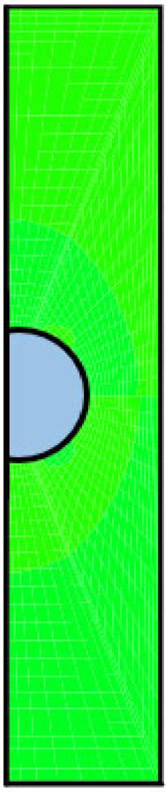

Figure S1. Two-dimensional physical domain.

The pseudo-spectral method can not be applied directly to this region, though, as the method requires an orthogonal geometric domain to lay out collocation points in each direction for further mathematical treatment and simplification [7]. Moreover, it is difficult to express in explicit form the precise locations of a cylindrical pore and the upstream or downstream planes in the corresponding spherical coordinates.

To reconcile with this mismatch of irregular domain boundaries problem, as well as the expression of the boundary location, the two-dimensional mathematical domain in Figure S1 is further decomposed into six separate subregions, together with the neighboring interior half-domain of the droplet, as shown in Figure S2.

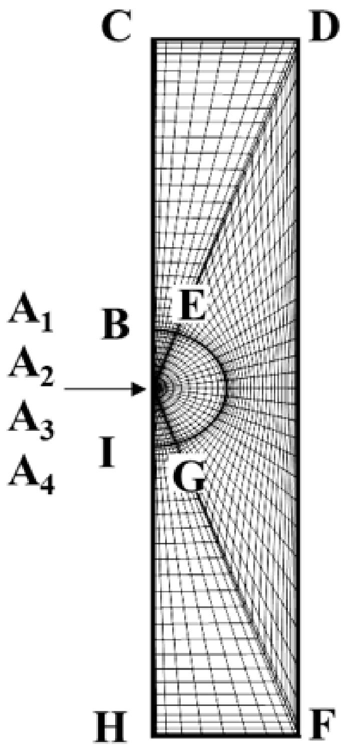

Figure S2. Mesh diagram of the sub-regions.

The above mesh diagram is then further transformed to the corresponding rectangular region (x, y), as shown below in Figure S3 via the conformal mapping technique. The details can be found elsewhere [8].

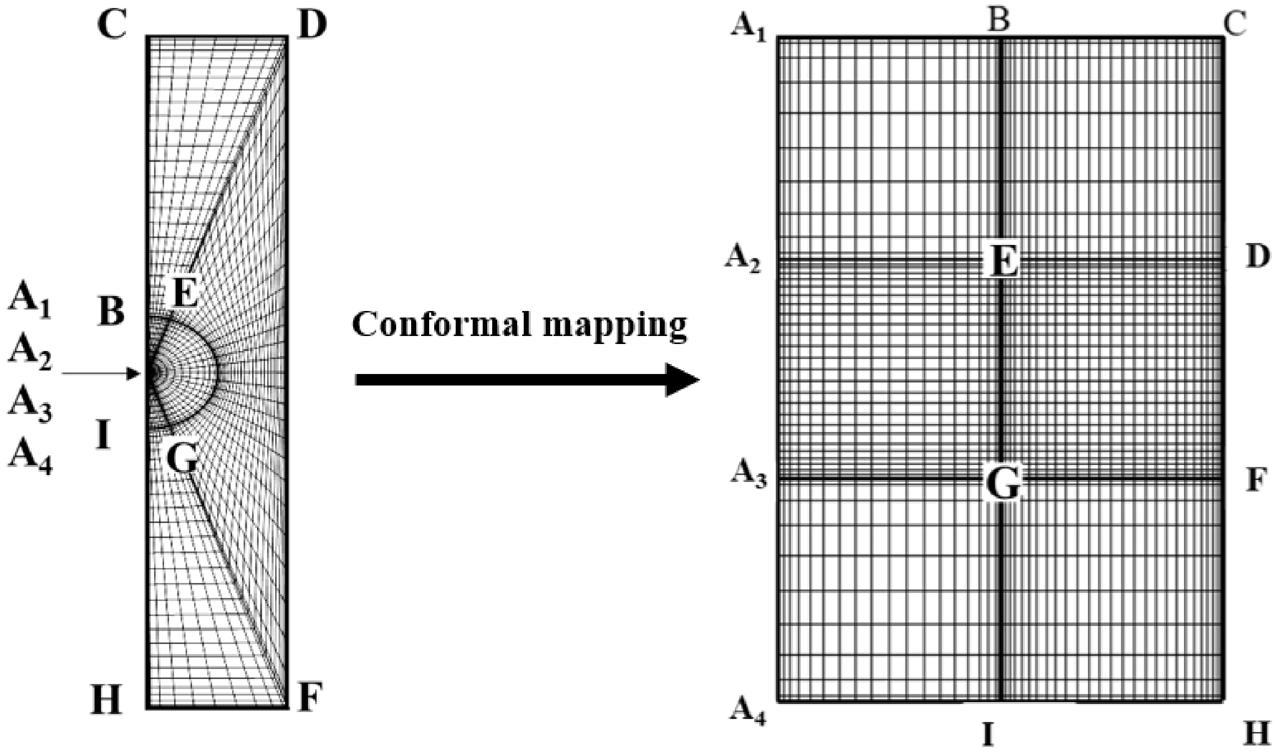

Figure S3. Orthogonal mesh diagram of the sub-regions after conformal mapping.

Figure S3 is the ultimate mathematical domain for computation with the pseudo-spectral method adopted in each subdomain, respectively, and the continuity of each

variable and its derivative is enforced at each interface of the three sub-regions shown. This type of treatment is referred to as the "patched pseudo-spectral method" [7], which is an extension of the original classic pseudo-spectral method. The details of this mapping technique can be found elsewhere [9].

C. A List of the Relative Permittivity of Various Materials

Table S1. Table of the relative permittivity (ε_r) of various materials [10].

Material (English name)	Chemical formula (alias)	ε_r
Water	H_2O	78.5
Formic acid	HCOOH	58.5
N,N-Dimethylformamide (DMF)	$HCON(CH_3)_2$	36.7
Methanol	CH_3OH	32.7
Ethanol	C_2H_5OH	24.5
Acetone	CH_3COCH_3	20.7
1-Hexanol (n-hexanol)	n-$C_6H_{13}OH$	13.3
Acetic acid	CH_3COOH	6.15
Benzene	C_6H_6	2.28
Carbon tetrachloride	CCl_4	2.24
n-Hexane	n-C_6H_{14}	1.88
n-Butane	n-C_4H_{10}	1.78
Air	—	1.005 3
Silicone oil	(polydimethyl-siloxane)	2.3 – 2.8
Polystyrene	—	2.4 – 2.6
Paraffin wax	—	2.0 – 2.1
Rubber (natural)	—	2 – 3
Wood (typical dry)	—	≈ 2.8

References

[1] Gottlieb D, Orszag SA. Numerical analysis of spectral methods: theory and applications. SIAM; 1977.

[2] Canuto C, Hussaini MY, Quarteroni A, Zang TA. Spectral methods: evolution to complex geometries and applications to fluid dynamics. Springer Science & Business Media; 2007.

[3] Lee E, Chu J-W, Hsu J-P. Electrophoretic mobility of a sphere in a spherical cavity. Journal of colloid and interface science. 1998;205:65-76.

[4] Lee E. Theory of electrophoresis and diffusiophoresis of highly charged colloidal particles. Academic Press; 2018.

[5] Hussaini MY, Zang TA. Spectral methods in fluid dynamics. 1986.

[6] O'Brien RW, White LR. Electrophoretic mobility of a spherical colloidal particle. Journal of the Chemical Society, Faraday Transactions 2: Molecular and Chemical Physics. 1978;74:1607-26.
[7] Orszag SA. Spectral methods for problems in complex geometrics. In: Numerical methods for partial differential equations. Elsevier; 1979. p. 273-305.
[8] Chang H-T. Electrophoretic Motion of Colloidal Particle in a Micro- or Nano- channel, ph.D. thesis, National Taiwan University. 2012.
[9] Huang C-H, Hsu H-P, Lee E. Electrophoretic motion of a charged porous sphere within micro-and nanochannels. Physical Chemistry Chemical Physics. 2012;14:657-67.
[10] Rumble J. CRC handbook of chemistry and physics. CRC press Boca Raton, FL; 2017.
